# The fourth vaccination with a non-SARS-CoV-2 variant adapted vaccine fails to increase the breadth of the humoral immune response

**DOI:** 10.1038/s41598-023-38077-x

**Published:** 2023-07-04

**Authors:** Sascha Hein, Catarina Sabino, Nuka Ivalu Benz, Esra Görgülü, Thorsten Jürgen Maier, Doris Oberle, Eberhard Hildt

**Affiliations:** 1 Department of Virology, Paul-Ehrlich-Institut, Paul-Ehrlich Street 51-59, 63225 Langen, Germany; 2 Division of Pharmacovigilance, Paul-Ehrlich-Institut, Paul-Ehrlich Street 51-59, 63325 Langen, Germany

**Keywords:** Immunology, Diseases

## Abstract

Escape mutations in the spike protein of SARS-CoV-2 are a major reason for Omicron breakthrough infections. After basal vaccination only very low titers of Omicron neutralizing antibodies are present. However, booster vaccinations induce higher titers against the Omicron variant. The neutralization of the Delta and Omicron variants by sera obtained 6 months after 3rd vaccination and 2 weeks or 6 months after 4th vaccination with a monovalent RNA vaccine (Spikevax) was analyzed. It was observed for the Omicron variant that 6 months after the fourth vaccination, the titer returns to the same very low neutralizing capacity as 6 months after the third vaccination. The Delta variant neutralizing capacity wanes with a comparable kinetic although the titers are higher as compared to the Omicron variant. This indicates that the fourth vaccination with a monovalent vaccine based on the ancestral isolate neither affects the kinetic of the waning nor the breadth of the humoral response.

## Introduction

For the past year, the Omicron variant has been circulating and dominating the infection landscape within the COVID-19 pandemic^1^. The Omicron variant, its subvariants BA.1, BA.2, BA.5 and more recently, BQ.1.1, BA.2.75.1 and XBB/XBB.1 are characterized by an enormous escape potential due to destruction/deletion of a variety of epitopes recognized by neutralizing antibodies^2,3^. Therapeutic monoclonal antibodies are hardly effective especially against the recent Omicron variants. Moreover, the number of breakthrough infections in vaccinated individuals has significantly increased since the emergence of the Omicron variant^4–6^. Due to the numerous mutations in the Omicron spike protein, which mediates the entry into the cell by binding to the human ACE2 receptor, many of the antibodies elicited by vaccination and/or infection fail to bind to the mutated spike and thereby cannot exert their neutralizing potential^7^. Under these conditions, efficient neutralization of the Omicron variants by the remaining neutralizing antibodies requires high affinity and titers. Booster vaccinations are given for several reasons. The antibody titer rises rapidly within the first 2 weeks after a booster vaccination, providing the best protection against the virus, but drops back to a baseline level within the first few months^8^. This baseline antibody level is built up by the immunologic memory. Immunological memory should also be triggered by booster vaccination, which increases memory B and long-lived plasma cells^9^. Furthermore, booster vaccination stimulates a broader immune response formed by somatic hypermutation and antibody affinity maturation^10,11^. In the case of mRNA vaccination, Paul Naaber's study described that the decrease of the neutralizing titers after booster vaccination occurs more slowly as compared to the titer after two vaccinations, indicating immunological memory and a positive long-term effect of the third vaccination^12^. In Germany, the fourth vaccination (second booster) has been recommended for certain groups at risk since February 2022^13^. The recommendation was based on a monovalent non-adapted vaccine. This raises the questions (1) whether the second booster has a further impact on the breadth of the humoral immune response and (2) whether a longer persisting humoral immune response can be induced.

## Results

### Study design

Healthcare workers who had received the third vaccination 6 months before were recruited for this study. This time point corresponds to the first blood collection (6m3V). At this time, subjects received the fourth vaccination (second boost). Two weeks (2w4V) and 6 months (6m4V) after this vaccination, blood samples were collected again. None of the study participants had a previous SARS-CoV-2 infection. The first vaccinations were performed with the original vaccine BNT162b2. Participants were vaccinated for the fourth time in mid-February 2022. At that time, no Omicron-matched vaccines were available. However, studies have revealed that a half dose of Spikevax as a fourth vaccination results in a higher titer and better cellular response than a full dose of BNT162b2^14^. Therefore, the fourth vaccination was performed with 50 µg Spikevax (Moderna).

### Similar antibody titers 6 months after 3rd and 6 months after 4th vaccination

**Figure 1:**
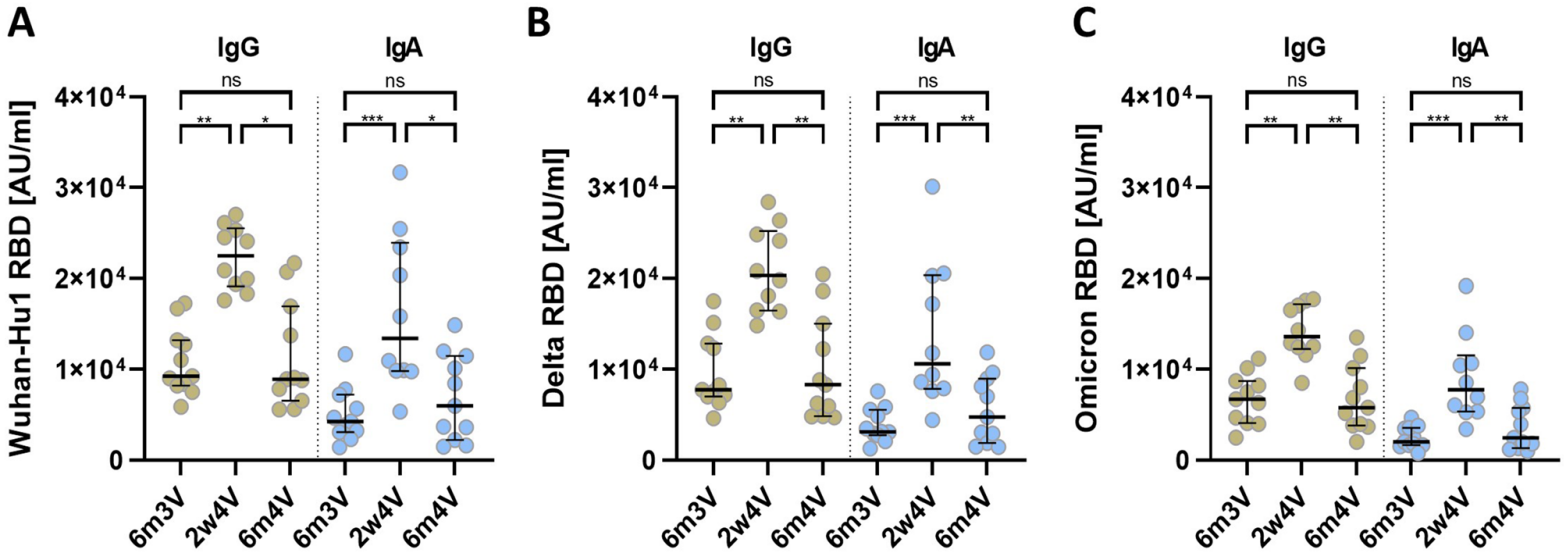
IgG and IgA antibody responses of vaccinated individuals. Anti-SARS-CoV-2 RBD IgG and IgA antibody levels against different SARS-CoV-2 variants at 6 months after third vaccination (6m3V), 2 weeks after fourth vaccination (2w4V) and 6 months after fourth vaccination (6m4V). The median with interquartile range is shown for each time point. (**A**) Antibody response against the original Wuhan-Hu1 SARS-CoV-2. (**B**) Antibody response against the Delta variant of SARS-CoV-2. (**C**) Antibody response against the Omicron variant of SARS-CoV-2. p values are based on the Kruskal–Wallis test with FDR correction: > 0.0331 (ns); 0.0331 (*); 0.0021 (**); 0.0002 (***); < 0.0001 (****).

Antibody levels (Fig. 1, Table 1) against the SARS-CoV-2 Wuhan-Hu1 RBD protein before the fourth vaccination (6 months after the third vaccination, 6m3V) were still high for IgG [median: IgG 9243 AU/ml (IQR 8306–12,982 AU/ml)] and in a similar range of previously published data form Paul Naaber^12^. In addition to Naaber et al., we also analyzed the IgA level in the sera [IgA 4259 AU/ml (IQR 3166–6449 AU/ml)]. After the second boost (fourth vaccination, 2w4V) with Spikevax, the titers were significantly increased by 3.1-fold and 2.4-fold for IgA [median: 13,405 AU/ml (IQR 9852–22,645 AU/ml)] and IgG [median 22,487 AU/ml (IQR 19,553–25,107 AU/ml)], respectively. The titers at 6 months after the fourth vaccination (6m4V) were decreased for both subclasses to 5977 AU/ml (IQR 2912–10,787 AU/ml) (IgA) and 8918 AU/ml (IQR 7190–15,349 AU/ml) (IgG). Interestingly, the values 6 months after the fourth vaccination are not significantly different from the titers 6 months after the third vaccination, suggesting that the antibody repertoire at 6 months after the fourth vaccination is almost the same as before. Interestingly, the same pattern of increase and decrease is observed for titers against the SARS-CoV-2 Delta (IgG: 6m3V: 7739 AU/ml, 2w4V: 20,313 AU/ml, 6m4V: 8330 AU/ml; IgA: 6m3V: 3133 AU/ml, 2w4V: 10,620 AU/ml, 6m4V: 4735 AU/ml) and Omicron (IgG: 6m3V: 6717 AU/ml, 2w4V: 13,607 AU/ml, 6m4V: 5778 AU/ml, IgA: 6m3V: 2011 AU/ml, 2w4V: 7751 AU/ml, 6m4V: 2456 AU/ml) variants. Over all time points, titers against the two variants are lower compared to the ancestral virus. This indicates that 6 months after the fourth dose, (1) the Delta- and Omicron-specific titers persist on a lower level as compared to the Wuhan-specific titer, (2) the kinetics of antibody waning are the same for all variants, and (3) the antibody titer returns to the baseline value measured before the fourth vaccination for all SARS-CoV-2 variants tested.

**Table 1:** Summary of the measured data to the different time points.

	6m3V	2w4V	6m4V
Anti-S-RBD IgG antibodies (AU/ml; median/IQR)			
SARS-CoV-2 Wuhan-Hu1	9243/8306–12,982	22,487/19,553–25,107	8918/7190–15,349
SARS-CoV-2 Delta	7739/7099–12,605	20,313/16,925–24,667	8330/5395–13,638
SARS-CoV-2 Omicron	6717/4383–8453	13,607/12,473–16,928	5778/3913–9109
Anti-S-RBD IgA antibodies (AU/ml; median/IQR)			
SARS-CoV-2 Wuhan-Hu1	4259/3166–6449	13,405/9852–22,654	5977/2912–10,787
SARS-CoV-2 Delta	3133/2834–5199	10,620/8088–19,523	4735/2404–8560
SARS-CoV-2 Omicron	2011/1655–3524	7751/5535–10,632	2456/1570–5539
IgG binding ratio (%; median/IQR)			
Omicron vs. Wuhan-Hu1	60.8/52.4–65.3	64.9/58.7–67.9	59.9/55.8–65.2
Delta vs. Wuhan-Hu1	85.4/83.7 92.1	95.5/86.4 100.9	86.1/80.0–91.8
IgA binding ratio (%; median/IQR)			
Omicron vs. Wuhan-Hu1	49.3/42.5–64.9	55.0/51.9–61.3	52.5/47.2–67.3
Delta vs. Wuhan-Hu1	81.0/74.1–89.4	83.7/80.0–87.2	84.1/79.2–87.5
Neutralization (GMT)			
Delta	248	449	166
Omicron	47	421	46

### The second booster has no impact on the breadth of the spike-specific immune response

**Figure 2:**
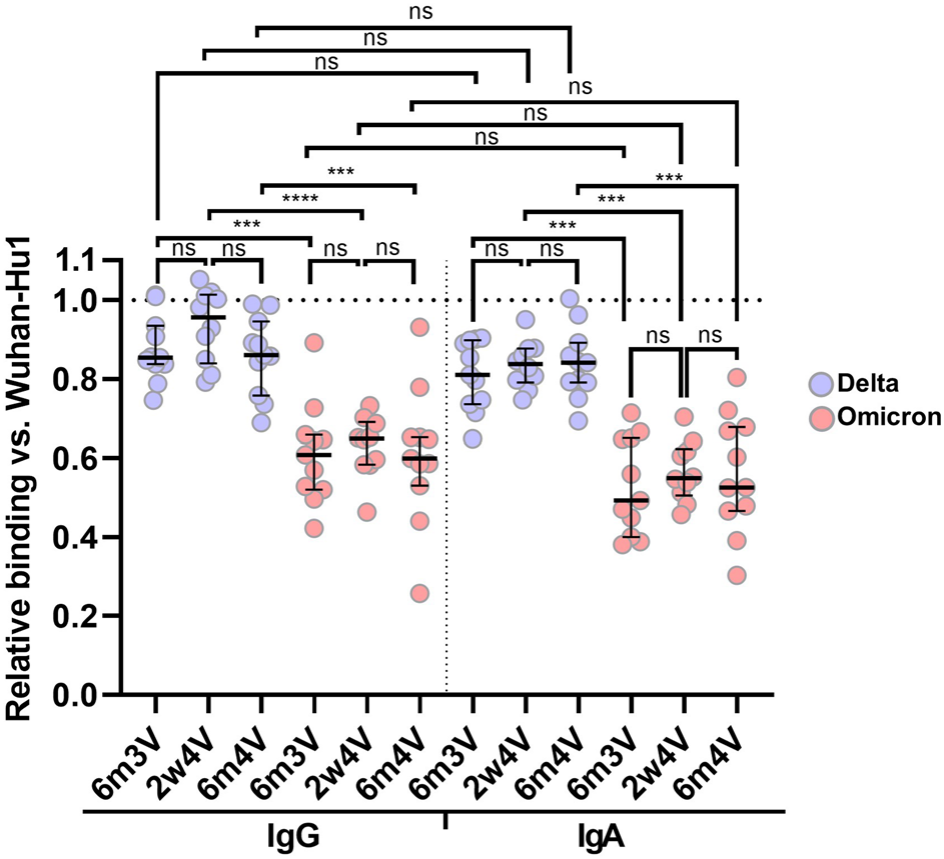
Relative binding of Anti-SARS-CoV-2 RBD antibodies against Delta and Omicron variant vs. Wuhan-Hu1. Relative binding ratio of SARS-CoV-2 variant vs. Wuhan-Hu1 for each time point and for the subclasses IgG and IgA at 6 months after third vaccination (6m3V), 2 weeks after fourth vaccination (2w4V) and 6 months after fourth vaccination (6m4V). The dotted line represents the ratio 1, which indicates no difference in binding to the wild-type virus Wuhan-Hu1. The median with interquartile range is shown for each time point. p values are based on the Kruskal–Wallis test with FDR correction: > 0.0331 (ns); 0.0331 (*); 0.0021 (**); 0.0002 (***); < 0.0001 (****).

To address the question, if a fourth vaccination will provide more long-lived antibodies and broaden the immune response against the SARS-CoV-2 Delta and Omicron variant, we investigated the binding of the antibodies to the respective variant and determined the relative binding ratio between the SARS-CoV-2 variants and the Wuhan-Hu1 wild-type strain (Fig. 2, Table 1). A decrease in the amount of bound IgG antibodies of 4.5–14.6% for the Delta Variant (for IgA 15.9–18.9%) and 35.1–40.1% for the Omicron variant (for IgA 45.0–47.5%) could be observed. Interestingly, it can be observed that at the time point 2w4V the relative binding of IgG antibodies in the case of the Delta variant increases from 85.4 to 95.5%. However, the difference is not significant according to Kruskal–Wallis-test (p value: 0.2991). This could indicate that only a few Delta-specific memory cells are boosted with the fourth vaccination, resulting in a marginal (not significant) increase in Delta-specific IgG titers 2 weeks after the fourth vaccination. Furthermore, the difference between the Omicron and the Delta variant are for all time points and both subclasses significant. However, within the SARS-CoV-2 variants we cannot measure any significant difference between the time points and Ig subclasses, respectively. This indicates that the breadth of the immune response is not further affected by the fourth vaccination.

### Fourth vaccination with an original Wuhan-Hu1 vaccine boosts the neutralization capacity against Omicron for a short time

**Figure 3:**
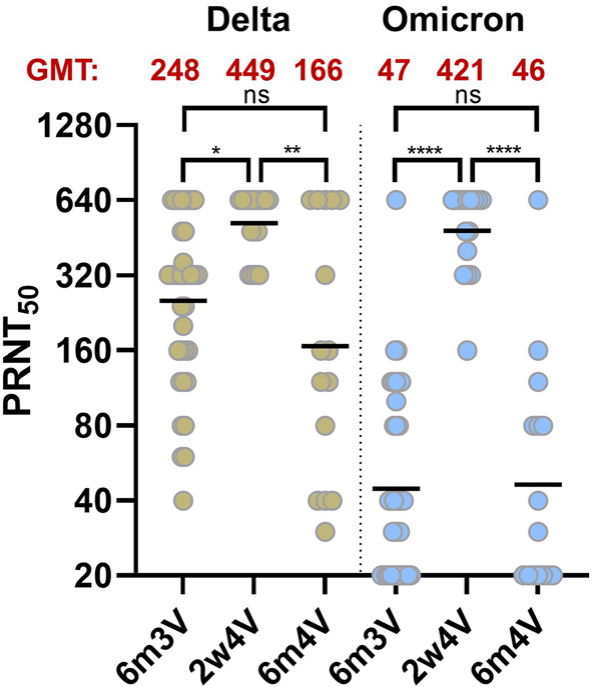
Neutralization capacity of vaccine-elicited sera against Delta and Omicron variant. Plaque reduction neutralization titer against the SARS-CoV-2 Delta and Omicron variants at 6 months after third vaccination (6m3V), 2 weeks after fourth vaccination (2w4V) and 6 months after fourth vaccination (6m4V). The assay was performed in duplicate and the neutralization is represented by the PRNT_50_ value. Blue bar represents the geometric mean. GMT; geometric mean titer. The neutralization data for time points 6m3V and 2w4V contains all initial subjects which are part of the cohort and are previously published^7^. p values are based on the Kruskal–Wallis test with FDR correction: > 0.0331 (ns); 0.0331 (*); 0.0021 (**); 0.0002 (***); < 0.0001 (****).

To validate the ELISA based data, PRNT_50_ assays with the Delta and Omicron variant of SARS-CoV-2 were performed (Fig. 3, Table 1). This neutralization assay reflects the entire neutralizing antibody repertoire of the sera, rather than just individual subclasses of antibodies. The titers against both variants after the second booster (2w4V; GMT: Delta: 449; Omicron: 421) were approximately equal. Nonetheless, 6 months after the fourth vaccination (6m4V; GMT: Delta: 166, Omicron: 46), these titers drop again to values similar to those at the 6m3V time point (GMT: Delta: 248, Omicron: 47). Thus, an identical trend is seen between the ELISA and neutralization assay. The decrease in the neutralization values to the level of the time point before the fourth vaccination indicates not only a decrease in titers, but also a similar antibody repertoire between the two time points. This shows at the level of neutralization capacity that (1) the fourth vaccination with a Wuhan-Hu1 vaccine causes a short-term increase of Omicron neutralizing antibodies, (2) does not further increase the breadth of the humoral immune response and (3) 6 months after vaccination there is no impact on in the baseline titer against the Omicron or Delta variant.

## Discussion

First, our data show that the sera collected prior the fourth and post the fourth vaccination have lower antibody levels against Omicron compared to the Delta or Wuhan-Hu1 variant. This finding is consistent with the literature^14,15^. In case of the IgG subclass, all sera showed an identical reduction in the titer of Omicron RBD-binding antibodies of approximately 38% compared to Wuhan-Hu1 RBD across all time points. Interestingly, this is not only the case for the IgG subclass, but also for the IgA subclass. After the fourth vaccination, a 2.5-fold increase in titer was measured which is within the range described^14^. However, the titer at 6m4V was back to the level before the fourth vaccination. The same results were measured with the plaque assay (Fig. 3), reflecting the neutralizing capacity. Here, the neutralization titer 6 months after the fourth vaccination was at the same level as before the fourth vaccination. This suggests similar kinetics of antibody waning and an almost identical antibody breadth after the third and fourth vaccinations. Paul Naaber et al*.* described that the first booster vaccination has a positive effect on immunological memory and results in an increase in the baseline titer^12^. Moreover, they observed that after the third vaccination the waning of the antibody titer is slower than after the second vaccination. With respect to the first and second booster vaccination (third and fourth vaccination) we observed similar titers at the time points 6m3V and 6m4V. Therefore, we hypothesize that there is no long-term (> 6 months) improvement in antibody/neutralization titers after the fourth vaccination with a wild-type vaccine. The fourth vaccination with the spike protein corresponding to the Wuhan-Hu1 isolate causes a stronger increase of titer of binding or neutralizing antibodies as compared to the third vaccination^16^. However, the titers decline to the same level 6 months after the second booster as at 6 months after the first booster. Only for a limited period after the vaccination there is a robust increase of antibody titers against Omicron. The observation that the titer drops 6 months after the second booster to the same level as 6 months after the first booster no further broadening of neutralizing and RBD-specific antibodies by somatic hypermutation and affinity maturation occurred which could be stimulated by a second boost. In terms of humoral immune response, the second booster vaccination with a wild-type vaccine against the current variants does not lead to a more sustained improvement than the first booster vaccination. Remarkably, natural infection prior to vaccination has been described to elicit a broader immune response against the new SARS-CoV-2 variants than vaccination alone^17^. As of September 01, 2022, the CHMP (Committee for Medicinal Products for Human Use) from the EMA (European Medicines Agency) has approved the first Omicron-adapted vaccines. Currently, bivalent adapted mRNA vaccines encompassing the Omicron BA.1 or BA.4/0.5 spike gene are approved. These Omicron-adapted vaccines could result in a broader immune response and expansion of immunologic memory as a second booster vaccine compared to the original vaccines based on the Wuhan-isolate. However, this requires that the novel sequences specific for the emerging Omicron variants with replaced epitopes recognized by neutralizing antibodies are immunogenic and trigger the formation of neutralizing antibodies binding with high affinity to the novel target structure. The large antigenic distance between the original sequence and the variant-specific sequence could mean that a robust induction of Omicron-specific antibodies requires a prime boost regimen. However, immunological imprinting (antigenic sin) could prevent robust formation of neutralizing antibodies specific for the “new” component of a bivalent vaccine and drive the immune response to the conserved epitopes. Here, a large antigenic distance between the original antigen (Wuhan) and the “new” antigen is desirable to overcome the limitations triggered by due antigenic sin. Due to the continuous emergence of new variants this aspect becomes more and more relevant. In light of this, the data provided here could be relevant for development of future vaccination strategies and evaluation of the future role of a monovalent vaccine.

Several limitations of our study need to be mentioned. To analyze the dynamics of the antibody titer, we used IgG- or IgA-specific ELISA assays. This ELISA assay detects only the RBD-binding antibodies and does not provide information about the antibodies that bind outside the RBD (in the whole spike protein). Therefore, the data do not reflect the total anti-spike-specific antibody titer. To compensate this, we also analyzed the neutralization titer by plaque assay, the gold standard method for neutralization assays^18^. However, this assay analyzes the total titer and not an Ig subclass specific titer. Furthermore, it could be argued that our sample size (n = 11) is too small to make such hypotheses. However, the anti-Wuhan-Hu1 IgG titer of our cohort 6m3V is in a comparable range to previously published cohorts^12^. It should be emphasized that analysis of antibody titers only represents a facet of the whole immune response and does not reflect analysis of T-cell response which plays an important role for prevention of severe COVID-19^19–21^.

## Methods

### Cell lines and virus strains

For the experimental work we used HEK239T (ATCC CRL-3216™) and Vero E6 (ATCC^®^ CRL-1586™) cells. Cell lines were cultivated in DMEM (in Dulbecco’s Modified Eagle’s medium, Sigma, Germany) medium supplemented with 1% penicillin/streptomycin, 1% l-glutamine and 10% fetal bovine serum (FBS Bio&SELL GmbH, Germany). Incubation were performed with 5% CO_2_ at 37 °C. The SARS-CoV-2 variants of concern B.1.1.617.2 (aka SARS-CoV-2 Delta variant; isolate hCoV-19/Germany/NW-RKI-I-2021) and B.1.1.529 (aka SARS-CoV-2 Omicron BA.1 variant; isolate hCoV-19/Netherlands/NH-EMC-1720/2021) as well as an ancestral isolate UVE/SARS-CoV-2/2020/FR/702 (Wuhan-HU1; GenBank: MT777677.1) were used. Strains were provided by the EVAg and the Robert Koch Institut. Infectious work was performed under biosafety level-3 (BSL-3) terms.

### SARS-CoV-2 RBD protein production

For the production of soluble RBD protein, the mutant *rbd* genes were cloned into the plasmid pCAGGS-sRBD (kindly provided by Florian Krammer, Icahn School of Medicine at Mount Sinai), which encodes for a RBD protein fused with a C-terminal hexa histag. The delta-*rbd* and omicron-*rbd* sequences were taken from the respective isolates, and the generation of the plasmids was previously described^7^.For the validation of all generated plasmids, sequencing was performed. HEK293T cells were grown in DMEM supplemented with 10% FCS, 1% Pen/Strep and 1% l-glutamine and seeded in 150 mm dishes with a cell density of 4.5 × 10^6^ cells/dish 6 h prior transfection. The transfection mix was prepared by mixing 15 µg plasmid DNA in 1.5 ml PBS with 90 µg of PEI in 1.5 ml PBS. After 16 h, medium was removed and 30 ml of fresh DMEM medium were added to the cells. At 72 h post transfection, the supernatant was harvested, sterile-filtered and mixed 1:1 with wash buffer (PBS supplemented with 40 mM imidazole, pH 8.0). A 5 ml Ni–NTA affinity column was equilibrated with 5 CV of wash buffer before loading with the diluted supernatant. The column was washed with 10 CV wash buffer, followed by elution of the protein with 250 mM imidazole in PBS. The buffer exchange with PBS was performed by centrifugation at 4 °C and 4000×*g* using a 10 kDa Amicon Centrifugal Filter.

### Study material and ethics

Our study (PEI-SARS-CoV2; 2020-1664_4-evBO) was approved by the ethics committee (Landesärztekammer Hessen, 60314 Frankfurt am Main). A written informed consent was obtained from all participants. The study was performed in compliance with the provisions of the Declaration of Helsinki from the World Medical Association and good clinical practice (GCP) guidelines. Overall, 11 subjects were recruited with a mean age of 46 (range 27–63). 4 subjects are men and 7 are women. From each subject, 10 ml of blood were collected in a BD Vacutainer SST II Advance (Becton Dickinson Rowa, Germany). After 30 min blood coagulation time at room temperature, the samples were centrifuged 10 min at 2000×*g* and 4 °C. The serum was aliquoted and stored at − 80 °C. After inclusion in the study, all participants were tested for anti-SARS-CoV-2 nucleocapsid antibodies to rule out previous infections. Actual infections were excluded by commercial rapid antigen test (Longsee 2019-nCoV Ag Rapid detection kit). The participants were subjected every 2 days to the tests.

### Plaque reduction neutralization test (PRNT50)

The protocol was adapted from Hein et al.^22^. The plaque reduction neutralization test was performed in duplicate. Vero E6 cells were seeded in 12-well plates with a cell density of 2.5 × 10^5^ cells/well and incubated for 24 h with 5% CO_2_ at 37 °C. Sera were serially diluted twofold (1:20 to 1:640) and incubated with 80 PFU (plaque forming units) of the different SARS-CoV-2 variants in a total volume of 100 µl/well at 37 °C for 1 h. After removing the cell culture medium and washing with PBS, DMEM complete without FCS was added to the cells. Subsequently, 100 µl of each dilution were added to the cells each and incubated for 1 h at 37 °C while shaking the plate regularly. Afterwards, the medium was removed from the cells and carefully replaced with pre-warmed DMEM complete mixed with a melted 4% liquid agarose solution in a 1/10 ratio. The plates were incubated at 37 °C with 5% CO_2_ and ≥ 90% humidity for 4 days after the agarose solidified. Afterwards, cells were fixed with 8% formaldehyde in PBS for 20 min at 37 °C. Next, the agarose and formaldehyde were removed and the cells were washed once with PBS. To visualize the plaques, 0,1% crystal violet (Sigma Aldrich) in 20% EtOH was added to the cells and incubated for 15 min at room temperature. The plaques were counted after removing the crystal violet solution and washing the cells with H_2_O.

### ELISA

To determine the specific binding of IgG and IgA to the RBD variants, an in-house ELISA was performed^23^. For evaluation of this in-house ELISA an international Standard from NIBSC was used (NIBSC Anti-SARS-CoV-2 Antibody Diagnostic Calibrant 20/162). 96-well plates were coated with RBD diluted in PBS with a concentration of 2 µg/ml and incubated at 4 °C overnight. After removing the coating solution, the plates were blocked with 10% FCS in PBS for 1 h at room temperature. The plates were washed with PBS-T (PBS supplemented with 0.05% Tween) three times between each step. Sera were prediluted 1:50 in 10% FCS in PBS and 50 µl of each dilution were added to the wells, followed by a 2 h incubation at room temperature. Afterwards, a 1:3000 dilution of HRP-linked Anti-human IgG antibody and of HRP-linked Anti-human IgG antibody was prepared in 10% FCS in PBS and 50 µl were added per well for 1 h. After three final washing steps with PBS-T, the plates were developed by adding 75 µl of TMB substrate solution to each well. After 5 min incubation, the reaction was stopped by the addition of 75 µl sulfuric acid. Detection of the optical density at 490 nm was measured in a Tecan reader. To calculate the relative binding of the different VOCs to Wuhan-Hu1, the mean of the IgG and IgA anti-Wuhan-Hu1 titers was set to 1, and the individual IgG and IgA anti-SARS CoV-2 variant titers were calculated in proportion to 1.

### Statistical analysis

Data Analysis was performed using Microsoft Excel 2019 and GraphPad Prism version 9. Statistical significances for ELISA experiments were calculated using Kruskal–Waals test. *p *values were FDR-adjusted with the Benjamini–Hochberg method. Only *p* values of 0.05 or lower were considered as statistically significant [p > 0.0331 (ns); 0.0331 (*); 0.0021 (**); 0.0002 (***); < 0.0001 (****)].

## Data Availability

All data described in this study are available from the corresponding authors (SH and EH) upon request.
